# A Case of Facial Neuralgia Cured by the Removal of a Tooth Containing Pulp Calculi

**Published:** 1895-08

**Authors:** D. S. Arnold

**Affiliations:** Atlanta, Ga.


### A CASE OF FACIAL NEURALGIA CURED BY THE REMOVAL OF A TOOTH CONTAINING PULP-CALCULI.

BY D. S. ARNOLD, M. D., D. D. S., ATLANTA, GA.

The patient, Mrs. W., aged 41 years, presented herself for examination of her teeth, stating that for five years she had been a sufferer from facial neuralgia of the right side. The pain was almost continuous, except when relieved by anodynes, and was located on the right side, over the superior maxillary. She had exhausted the list of remedies recommended by a number of competent physicians without more than transient relief with any. I directed a spray of ice water against each tooth in turn of the upper jaw, and when the jet touched the second right molar of the upper jaw, the patient screamed with pain. Though the tooth was sound, I extracted it, and on section found two pulp stones, the size of squirrel shot, occupying the entire nerve cavity. The stones were hard, smooth, and accurately fitted the pulp-cavity. The relief from the tic douloureux was immediate and continuous.

The formation of calculi in the tooth nerve cavity is a rare phenomenon, and the theory of their formation in this location does not differ from it in any other. It can readily be understood how the continuous pressure exerted on the walls of the cavity could be sufficient to account for the pain, and how the removal of this source of irritation could cause instantaneous relief.

---

MENSTRUATION IN A CHILD AGED SIX.—Jacobovitch related this case before the St. Petersburg Society for Diseases of Children. At the age of three she was brought before Jacobovitch for hemorrhages. The genitals were normal. A considerable amount of a milky fluid exuded from the breasts, which were of the size of hen's eggs. The child is now over six years of age. She menstruates several times yearly, especially in summer. Before the catamenia the child becomes ill-tempered; afterwards she has headaches with vertigo. She is very anæmic. The breasts are as large as small oranges; the labia minora and clitoris slightly hypertrophied.—*Boston Medical and Surgical Journal.*

